# Single-Cell RNA Sequencing Outperforms Single-Nucleus RNA Sequencing in Analyzing Pancreatic Cell Diversity and Gene Expression in Goats

**DOI:** 10.3390/ijms26083916

**Published:** 2025-04-21

**Authors:** Jie Cheng, Tianxi Zhang, Yan Cheng, Kefyalew Gebeyew, Zhiliang Tan, Zhixiong He

**Affiliations:** 1 National Engineering Laboratory for Pollution Control and Waste Utilization in Livestock and Poultry Production, South-Central Experimental Station of Animal Nutrition and Feed Science in Ministry of Agriculture, Hunan Provincial Engineering Research Center for Healthy Livestock and Poultry Production, Institute of Subtropical Agriculture, The Chinese Academy of Sciences, Changsha 410125, China; chengjie231@mails.ucas.ac.cn (J.C.); zhangtianxi20@mails.ucas.ac.cn (T.Z.); chengyan181@mails.ucas.ac.cn (Y.C.); kefe@isa.ac.cn (K.G.); zltan@isa.ac.cn (Z.T.); 2 College of Advanced Agricultural Sciences, University of Chinese Academy of Sciences, Beijing 100049, China; 3 Yuelushan Laboratory, Changsha 410125, China

**Keywords:** goats, pancreas, sequencing, cell type

## Abstract

The objective of this study was to determine whether single-cell RNA sequencing (scRNA-seq) or single-nucleus RNA sequencing (snRNA-seq) was more effective for studying the goat pancreas. Pancreas tissues from three healthy 10-day-old female *Xiangdong black goats* were processed into single-cell and single-nucleus suspensions. These suspensions were then used to compare cellular composition and gene expression levels following library construction and sequencing. Both scRNA-seq and snRNA-seq were eligible for primary analysis but produced different cell identification profiles in pancreatic tissue. Both methods successfully annotated pancreatic acinar cells, ductal cells, alpha cells, beta cells, and endothelial cells. However, pancreatic stellate cells, immune cells, and delta cells were uniquely annotated by scRNA-seq, while pancreatic stem cells were uniquely identified by snRNA-seq. Furthermore, the genes related to digestive enzymes showed a higher expression in scRNA-seq than in snRNA-seq. In the present study, scRNA-seq detected a great diversity of pancreatic cell types and was more effective in profiling key genes than snRNA-seq, demonstrating that scRNA-seq was better suited for studying the goat pancreas. However, the choice between scRNA-seq and snRNA-seq should consider the sample compatibility, technical differences, and experimental objectives.

## 1. Introduction

The goat pancreas plays a crucial role in both endocrine and exocrine functions, mirroring its functions in other mammals. The goat pancreas is composed of a complex array of cells: the endocrine compartment, including islet cells and pancreatic polypeptide cells, regulates blood glucose via hormone secretion [1]; the exocrine compartment consists of acinar cells and ductal cells, mainly secreting digestive enzymes [2,3]; and the presence of intermediate cells suggests potential cellular plasticity, enabling adaptation to metabolic demands [4]. Studies have shown that insufficient pancreatic amylase secretion is the key limiting factor in starch digestion efficiency in the small intestine of ruminants [5]. Given the pancreas’s crucial role in digestion and nutritional metabolism, researchers continue to investigate its regulatory mechanism in various approaches [6]. Currently, immunohistochemical and histochemical analysis [7], collagenase-based digestion and isolation [8], and immunoelectron microscopy [9] are among the widely used methods to identify goat pancreatic cells. Nevertheless, these conventional methods lack the resolution required for molecular and genetic-level analyses. Single-cell RNA sequencing (scRNA-seq) is widely used in pancreatic research to characterize the heterogeneous cellular composition and profile transcriptional activity at single-cell resolutions [10]. Numerous studies on human and mouse pancreata have reported pancreata cell type characteristics, heterogeneity between typical endocrine cell types, and the classification of subpopulations within cell types [6,11].

Despite its wide adoption, scRNA-seq has notable limitations. For example, scRNA-seq relies on mechanical and enzymatic cell dissociation, which leads to low cell yield and causes non-physiological cellular stress [12]. Amplification bias and sample loss further compromise the quality and accuracy [13,14]. In addition, scRNA-seq is limited to fresh tissues, significantly restricting its applicability in various scenarios [15]. To address these limitations, snRNA-seq technology has been developed [16]. It offers several advantages by using isolated cell nuclei for sequencing, including avoiding dissociation-induced stress responses, accommodating frozen specimens, and capturing pre-spliced mRNA (introns + exons), revealing transcriptome states before cell sampling [12,17,18]. However, snRNA-seq is not universally superior. Studies indicated that snRNA-seq may require more complex protocols and equipment, potentially increasing the cost and experimental time [19], and it may underperform scRNA-seq in detecting low-abundance transcripts [18]. In practice, scRNA-seq remains the gold standard for cancer research [20], stem cell biology [21], and neuroscience [22], providing high-resolution insights into heterogeneity and molecular mechanisms. Conversely, snRNA-seq excels with complex or preserved tissues, such as brain samples [19], where traditional cell dissociation is impractical. Therefore, each method has distinct strengths and limitations. Selection should align with research objectives and sample constraints.

Although parallel comparative studies have validated snRNA-seq as a reliable alternative to scRNA-seq [23], pancreatic endocrine cell composition and organization differ significantly across mammalian species [24]. Notably, the goat pancreas shows distinct endocrine cell immunopositivity patterns and spatial distribution compared with closely related species like sheep [7]. To date, no study has systematically compared the efficacy of scRNA-seq and snRNA-seq in the goat pancreas. Therefore, the purpose of this study was to determine the optimal method for studying the goat pancreas by comparing the identification of cells and genes in scRNA-seq and snRNA-seq results.

## 2. Results

### 2.1. Raw Data Quality Statistics

**Figure 1:**
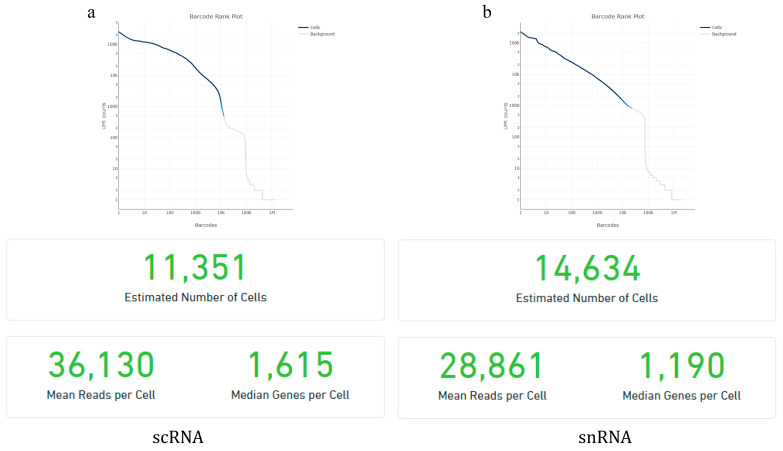
Quality control and quantitative results of scRNA-seq (**a**) and snRNA-seq (**b**).

The raw data from goat pancreas scRNA-seq and snRNA-seq were analyzed using Cell Ranger, version 6.1.2, the official analysis software of 10× Genomics, to assess data quality. The results of the sample quality control and quantification are shown in Figure 1. The scRNA-seq analysis identified 11,351 valid cells with an average of 36,130 reads per cell and a median of 1615 genes detected per cell. The snRNA-seq analysis identified 14,634 valid cells with an average of 28,861 reads per cell and a median of 1190 genes detected per cell. Overall, both datasets met quality thresholds, supporting their use in downstream bioinformatics analyses. Further analysis revealed that 24.2% of reads in snRNA-seq matched the antisense strand of the gene. The proportion of reads that were also aligned to the reference gene and originated from high-quality cells was lower at 53% (Table 1).

### 2.2. Comparison of Cell Annotations and Cell Type Ratio Statistics

After removing the low-quality cells, the expression data were normalized using the log normalize method in the “Normalization” function of Seurat software, version 4.4.0. The single-cell subpopulations were classified based on expression levels, and the classification results were further visualized using tSNE (t-distributed stochastic neighbor embedding) for nonlinear dimensionality reduction. The cell types and marker genes of the pancreas were collected from the published literature and a cell marker database (Cell Marker, http://117.50.127.228/CellMarker/, accessed on 15 July 2024), and the clustered goat pancreatic cells were then annotated based on the expression of marker genes identified in scRNA-seq and snRNA-seq.

**Figure 2:**
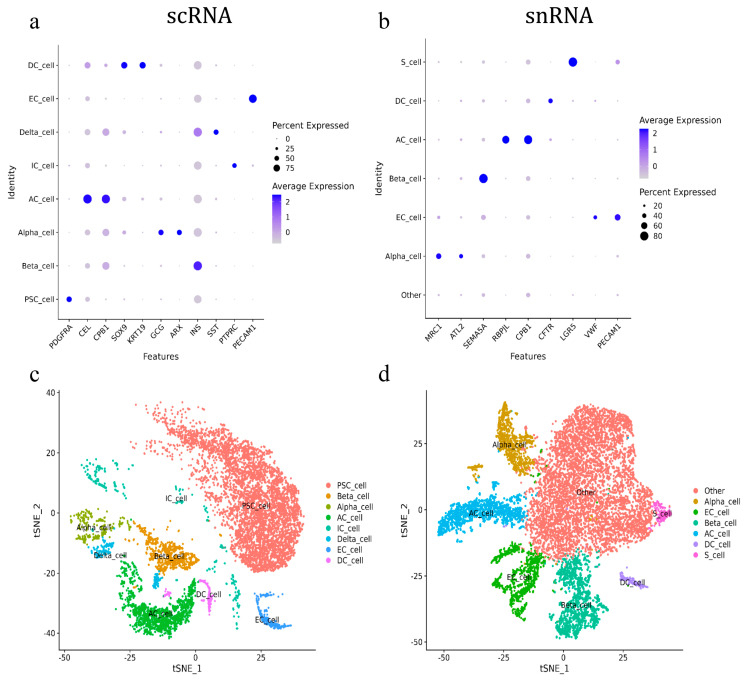
Annotation of goat pancreatic scRNA-seq and snRNA-seq cells. (**a**) ScRNA-seq marker gene score. (**b**) SnRNA-seq marker gene score. (**c**) ScRNA-seq cell subgroup classification tSNE. (**d**) SnRNA-seq cell subgroup classification tSNE.

The annotation results indicated that only pancreatic acinar cells (AC_cell) and endothelial cells (EC_cell) could be consistently annotated across both scRNA-seq and snRNA-seq using the canonical marker genes, CPB1 and PECAM1, respectively. The pancreatic acinar cells were also annotated using CEL and RBPJL in scRNA-seq and snRNA-seq, respectively. All other cell types were annotated by different marker genes. For scRNA-seq, pancreatic beta cells (Beta_cell), delta cells (Delta_cell), stellate cells (PSC_cell), and immune cells (IC_cell) were annotated using INS, SST, PDGFRA, and PTPRC, respectively. The ductal cells (DC_cell) were annotated by SOX9 and KRT19, while alpha cells (Alpha_cell) were annotated using GCG and ARX (Figure 2a). For snRNA-seq, pancreatic ductal cells, beta cells, and stem cells were annotated using CFTR, SEMA5A, and LGR5, respectively, while alpha cells were annotated by MRC1 and ATL2. Additionally, a large number of cells in snRNA-seq that lacked clear marker genes for identification were defined as other cells (Figure 2b). As a result, eight and six types of pancreatic cells were annotated in scRNA-seq and snRNA-seq, respectively. Pancreatic acinar cells, ductal cells, alpha cells, beta cells, and endothelial cells were annotated using both methods. Pancreatic stellate cells, immune cells, and delta cells were annotated exclusively in scRNA-seq, while pancreatic stem cells (S_cells) were annotated exclusively in snRNA-seq (Figure 2c,d).

Further analysis of the annotated cell types showed that scRNA-seq primarily identified pancreatic stellate cells, which accounted for 63.09% of the total cell population. These were followed by pancreatic follicular cells (16.38%), beta cells (8.13%), endothelial cells (2.9%), alpha cells (2.6%), ductal cells (2.08%), immune cells (2.81%), and delta cells (2%). In contrast, the snRNA-seq analysis revealed that the largest proportion of cell populations (56.18%) was classified as “other cells” due to a lack of specific annotation. The remaining cell types in snRNA-seq included pancreatic acinar cells (11.49%), beta cells (11.96%), endothelial cells (9.04%), alpha cells (8.15%), ductal cells (1.62%), and pancreatic stem cells (1.55%) (Table 2).

### 2.3. Analysis of scRNA and snRNA-Seq Goat Pancreatic Acinar Cells

**Figure 3:**
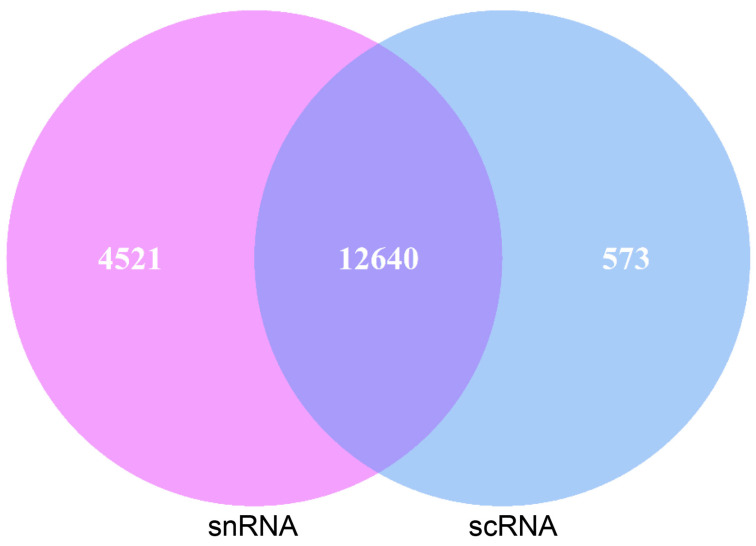
Gene statistics of scRNA-seq and snRNA-seq goat pancreatic acinar cells.

Pancreatic acinar cells were annotated with the same marker genes in both sequencing methods, allowing for a comparison of the genes detected in these annotated cells. Of the genes detected in pancreatic acinar cells, 12,640 genes were found in both scRNA-seq and snRNA-seq. Additionally, 573 genes were uniquely detected in scRNA-seq acinar cells, while 4521 genes were uniquely detected in snRNA-seq (Figure 3).

**Figure 4:**
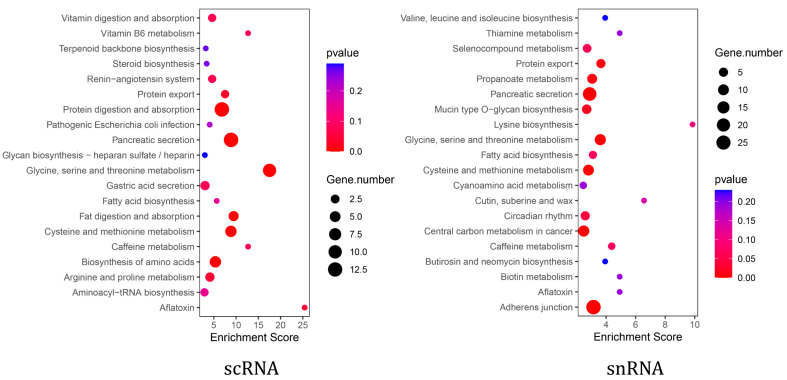
The scRNA-seq and snRNA-seq pancreatic acinar cell pathway enrichment.

KEGG enrichment analysis showed that pancreatic acinar cells from both scRNA-seq and snRNA-seq were significantly enriched in the pancreatic secretion pathway (Supplementary Table S1). Furthermore, scRNA-seq acinar cells exhibited additional enrichment in pathways related to exocrine pancreatic secretion, including protein digestion and absorption, as well as fat digestion and absorption (Figure 4). To further investigate functional differences, we analyzed the expression of digestive enzyme-related genes within the pancreatic secretion pathway in both scRNA-seq and snRNA-seq datasets. The results showed that the expression levels of genes, including *CEL*, *CPB1*, *CTRC*, *PNLIPRP2*, and *PRSS2*, were higher in scRNA-seq than in snRNA-seq (Table 3).

## 3. Discussion

Single-cell sequencing technology preserves the integrity of cellular nucleic acids, addressing the challenge of extracting high-quality nucleic acids from pancreatic tissue to some extent [25]. However, scRNA-seq and snRNA-seq impose different requirements on library construction for samples [26]. Due to the inherent instability of the pancreas tissue, the optimal sequencing method for preserving biological information remained unclear. Ideally, the chosen protocol should preserve the RNA integrity and cellular composition of the goat pancreatic tissue [27]. We, therefore, performed parallel scRNA-seq and snRNA-seq on goat pancreatic tissues to systematically compare their performance in capturing biologically relevant information.

After quality control of the downstream data, we compared the number of valid cells detected by the two methods, the average number of reads per cell, and the median number of genes detected per cell between the two methods. The results showed that both methods met the threshold quality for single-cell transcriptome analysis, although snRNA-seq showed limitations [28]. The snRNAseq showed a low reference genome alignment rate even in high-quality cells, suggesting a limited representation of goat pancreas cells from the snRNA-seq. Furthermore, the high proportion of reads aligned to the antisense strand suggested that snRNA-seq may provide limited information about the goat pancreas [19]. Possible reasons for these results were the presence of high levels of environmental RNA and the large number of cells with low RNA content during snRNA-seq nucleus extraction [16]. Therefore, given the quality of the sequencing data, scRNA-seq was more suitable for analyzing goat pancreas.

Both scRNA-seq and snRNA-seq successfully identified the major pancreatic cell types, such as acinar cells, ductal cells, alpha cells, and beta cells. These results demonstrated that both techniques were suitable for investigating the exocrine and endocrine functions in the goat pancreatic tissue [29]. However, statistical analysis of the annotated cell numbers revealed a discrepancy with previous knowledge of the pancreas, which demonstrated that follicular cells comprised about 70% of pancreatic tissue [30]. In contrast, this study found that the largest proportion of cells identified by scRNA-seq were pancreatic stellate cells, while 56.18% of cells in snRNA-seq could not be classified into a specific cell type. Single-cell transcriptome sequencing technology, which identifies cells based on the expression of marker genes [31], is influenced by numerous factors. These factors can affect the differential expression of both marker and non-marker genes, potentially impacting the resolution of cellular annotation [32]. Furthermore, the identification of cells in single-cell transcriptome sequencing is also influenced by human subjectivity during the determination of marker genes [33], which introduces artificial variability and may contribute to inconsistencies in cell annotation. In the present study, even when marker genes were highly and specifically expressed in their corresponding cells, differences in marker gene expression between the two sequencing methods were observed, likely due to differential expression of patterns in the same cells [34]. Furthermore, cellular annotation varied between scRNA-seq and snRNA-seq. For example, the pancreatic stellate cells, immune cells, and delta cells were uniquely annotated using scRNA-seq, while pancreatic stem cells were exclusively annotated using snRNA-seq. This highlights the significant discrepancies between the two methods in detecting marker gene expression across different cell types. In terms of the types of cells, scRNA-seq clearly annotated eight types of cells, while snRNA-seq identified six classes of cells, leaving a large number of cells undefined. This indicated that scRNA-seq offered higher resolution for goat pancreatic cells, providing a more comprehensive reflection of biological information in goat pancreas. Meanwhile, regarding the pancreatic exocrine function, scRNA-seq identified a higher number of pancreatic follicular cells compared with snRNA-seq.

As the primary functional unit of the pancreatic exocrine compartment [35], acinar cells secrete enzymes that are indispensable in the digestion of proteins, carbohydrates, and lipids [36]. Given the importance of pancreatic exocrine function, we compared scRNA-seq and snRNA-seq data from pancreatic acinar cells to determine the optimal single-cell transcriptome sequencing method for studying this function [37,38]. First, we compared genes detected by scRNA-seq and snRNA-seq in goat pancreatic acinar cells. Although snRNA-seq detected more genes than scRNA-seq in goat pancreatic acinar cells, previous analyses suggest that ambient RNA contamination during nuclear extraction may confound these results [39]. Therefore, the biological relevance of detecting more genes with snRNA-seq remains unclear. Moreover, while snRNA-seq is generally comparable to scRNA-seq, it may lack the equivalent sensitivity for detecting specific transcripts [18]. For this reason, we performed gene enrichment analysis for the detected gene in acinar cells using the two methods. Although both methods enriched the pancreatic secretory pathway, scRNA-seq identified more pathways related to the exocrine function, such as protein digestion and absorption and fat digestion. In contrast, genes identified using snRNA-seq analysis enriched in pathways associated with core cellular functions, including adherens junction, carbon metabolism, and amino acid metabolism. These suggested that while snRNA-seq detected more genes, it captured less biological information specifically relevant to pancreatic exocrine function. Furthermore, a comparative analysis of genes associated with the pancreatic secretion pathway and pancreatic digestive enzymes showed consistently higher expression levels in scRNA-seq than in snRNA-seq.

Our results—spanning sequencing data quality, cell identification numbers, and key gene expression levels—indicate that scRNA-seq may offer distinct advantages over snRNA-seq for caprine pancreatic research. However, these findings must be interpreted cautiously due to potential confounding factors, including limited sample size and technical biases inherent in sample processing. Specifically, the precision of both scRNA-seq and snRNA-seq depends critically on the number of cell capture and input RNA integrity. Sequencing an adequate number of high-quality cells per cell type is essential to minimize technical noise and improve expression quantification accuracy [40]. The reliance on extensive amplification in single-cell sequencing may introduce artifacts, including uneven coverage and quantification inaccuracies, owing to the susceptibility of low-input RNA degradation or contamination [41]. While sample multiplexing can lower costs, it may introduce batch effects, such as preparation methods or reagent-specific biases, potentially confounding biological interpretations [42]. Additionally, the modest sample size and inter-sample variability in this study may limit the generalizability of our conclusion. Thus, further studies should systematically compare both methods across diverse sample conditions (e.g., fresh versus frozen tissues) to validate their complementary utilities in caprine pancreatic research. Given the technical distinctions between scRNA-seq and snRNA-seq—including sample compatibility and detectable biological variations—an integrated application rather than the exclusive use of either may optimize caprine pancreas studies.

## 4. Materials and Methods

### 4.1. Reagent Pre-Preparation

For the preparation of the 10× cleaning solution, a 100× penicillin mixture (Solarbio, Beijing, China) was added to 500 mL of phosphate buffer PBS (Gibco, Grand Island, NY, USA), diluting the penicillin mixture to 10× concentration, which was referred to as the 10× cleaning solution. The prepared 10× cleaning solution was divided into two parts for external and internal laboratory use. The external portion was used to clean the pancreatic tissue during sampling, while the internal portion was used for repeated cleaning of pancreatic tissue during digestion and dissociation.

For the preparation of the 10× transfer solution, a 100× penicillin mixture was added to 50 mL DMEM/F12 medium (Gibco, Grand Island, NY, USA), diluting the penicillin mixture to a 10× concentration, which was referred to as the 10× transfer solution. After sampling, the pancreatic tissue was transported to the cell culture chamber in the transfer solution, which served as a protective agent. It was prepared prior to the test and placed on ice throughout its use.

### 4.2. Sample Collection

**Figure 5:**
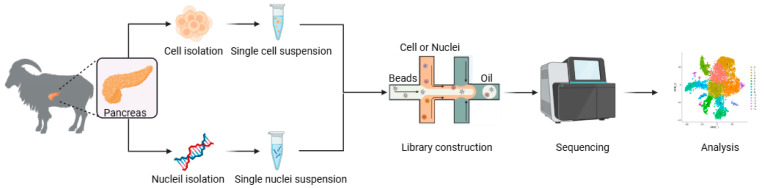
Workflow of scRNA-seq and snRNA-seq.

The experimental procedures of this study were conducted according to the guidelines of the Animal Ethics Committee of the Institute of Subtropical Agriculture, Chinese Academy of Sciences (CAS) (20200031). Three 10-day-old and healthy female *Xiangdong black goats* were fasted for at least 4 h prior to euthanasia. Immediately following exsanguination, the abdominal cavity was opened to expose the pancreas. The caprine pancreas, located within the mesoduodenum, displays a lobulated morphology extending from the stomach’s greater curvature to the spleen. Pancreata were rapidly dissected along their margins using sterile surgical scalpels with continuous irrigation using an ice-cold 10× PBS washing solution. The blood vessels and fascia were carefully removed from the samples before they were repeatedly washed 4 to 5 times with a 10× BPS cleaning solution. The samples were then stored in a pre-prepared 10× transfer solution and placed on ice during transport back to the laboratory. Then, the random number method was used to randomly select two pancreata for the scRNA-seq, and another pancreas was used for RNA-seq (Figure 5).

### 4.3. Sample Preparation for Goat Pancreas scRNA-Seq

For whole-cell dissociation, the pancreas was transported to the laboratory and digested with 1 mL of 2 mg/mL streptavidin at room temperature (approximately 22 °C) for a period of time. After digestion, the streptavidin solution was removed, and the tissue was incubated with 1% fetal bovine serum (FBS) (Gibco, Grand Island, NY, USA). After that, the tissue was washed twice with the same solution, and then the sample was ground using fire-polished glass pipettes with gradually decreasing pore sizes (600, 300, and 150 μm). The cell suspension was incubated on ice, and 4′-6-diamidino-2-phenylindole (DAPI) (Beyotime, Haimen, Jiangsu, China) was added to label dead cells (DAPI+) and live cells (DAPI-). The suspension was then filtered through a fine pore cell filter to remove cell aggregates. The cells were classified by excluding DAPI-positive events and fragments, and gates were set to include red fluorescence events (tdTomato-positive cells). Individual cells were collected into tubes containing 11.5 μL of collection buffer (SMART Seq v4 lysis buffer 0.83× #634894, Clontech, Mountain View, CA, USA), RNase inhibitor (0.17 U/μL), and ERCC (external RNA control consortium, final diluted MIX1) (1 × 10^−8^). After sorting, the tubes containing single cells were briefly centrifuged and stored at −80 °C.

Following the manufacturer’s recommendations, dissociated cells were treated with red blood cell lysis buffer and MACS dead cell removal kit (Miltenyi Biotec, Bergisch Gladbach, NRW, Germany) to remove red blood cells, dead cells, and cell debris. Afterward, the cells were counted using a hemocytometer (Thermo Fisher Scientific, Waltham, MA, USA) and examined for cell viability using trypan blue (Thermo Fisher Scientific, Waltham, MA, USA). Finally, all single-cell suspensions of each sample were free of cell debris and had high viability (over 90%). A volume sufficient to capture 8000 cells was calculated, and the cells were then further diluted to user guide concentrations (700–1200 cells/μL) using 1× PBS containing 0.04% BSA (Yita, Beijing, China) for 10× genomics sequencing.

### 4.4. Sample Preparation for Goat Pancreas snRNA-Seq

For nuclei isolation, the pancreas was transferred to a microcentrifuge tube, snap-frozen in a slurry of dry ice and ethanol, and stored at −80 °C until use. To isolate nuclei, frozen tissue was placed in homogenization buffer consisting of 10 mM Tris pH 8.0, 250 mM sucrose, 25 mM KCl, 5 mM MgCl_2_, 0.1% Triton-X 100 (Sigma, St. Louis, MO, USA), 0.5% RNasin Plus RNase Inhibitor (Promega, Madison, WI, USA), 1× protease inhibitor (Promega, Madison, WI, USA), and 0.1 mM DTT. Tissues were placed in a 1 mL Dounce homogenizer (DWK Life Sciences, Shanghai, China) and homogenized by gently tapping 10 times with a loose Dounce pestle, followed by 10 taps with a tight pestle to release the nuclei. The homogenate was filtered through a 30 μm cell strainer (Miltenyi Biotec, Bergisch Gladbach, NRW, Germany) and centrifuged at 900× *g* for 10 min to precipitate the nuclei. Nuclei were then resuspended in a staining buffer containing 1× PBS supplemented with 0.8% nuclease-free BSA and 0.5% RNasin Plus RNase inhibitor. Mouse anti-NeuN antibody (EMD Millipore, MAB377, Clone A60, Merck Millipore, Billerica, MA, USA) was added to the nuclei at a final dilution of 1:1000, and the nuclei suspension was incubated at 4 °C for 30 min. The nuclear suspension was then centrifuged at 400× *g* for 5 min and resuspended in fresh staining buffer (1× PBS, 0.8% BSA, 0.5% RNasin Plus). The secondary antibody (goat anti-mouse IgG (H+L) (Yeasen, Shanghai, China), Alexa Fluor 594 coupler, Thermo Fisher Scientific, Waltham, MA, USA) was applied to the nucleus suspension at a dilution of 1:5000 for 30 min at 4 °C. After incubation with the secondary antibody, the nuclear suspension was centrifuged at 400× *g* for 5 min and resuspended in a clean staining buffer. Prior to FACS, DAPI was applied to the nuclear suspension at a final concentration of 0.1 μg/mL, and the nuclear suspension was filtered through 35 μm nylon mesh to remove aggregates. Single nuclei were captured by gating DAPI-positive events to exclude debris and double peaks and then gated for Alexa Fluor 594 (NeuN) signal (Invitrogen, Eugene, OR, USA). Strips of tubes containing FACS-separated mononuclei were then briefly centrifuged and frozen at −80 °C.

### 4.5. Library Construction and Sequencing

Briefly, cell or nucleus suspensions were loaded into the latest 10× Chromium™ Single Cell 3′ Solution System (10× Genomics, Pleasanton, CA, USA) to generate single-cell gel-emulsion of beads (GEM). The scRNA-seq libraries were constructed using the 10× Genomics 3′ Kit v3 and quality checked using Agilent Bioanalyzer High Sensitivity Microarrays (Agilent Technologies, Santa Clara, CA 95051, USA). To minimize batch effects, libraries were constructed using the same version of the kit and following the same protocol. LC-Bio Technology Co., Ltd. (Hangzhou, China), sequenced the libraries on the same Illumina NovaSeq 6000 sequencing system (Illumina, San Diego, CA, USA)using a 150 bp pair-ended sequencing with a minimum depth of 20,000 reads per cell.

### 4.6. Analysis of Sequencing Data

The sequencing results were converted to FASTQ files using Illumina bcl2fastq software (version 5.01). The raw data were multiplexed, barcoded, and mapped to the goat reference genome (https://www.ncbi.nlm.nih.gov/assembly/GCF_001704415.1/, accessed on 18 July 2024) using the Cell Ranger package (version 3.1.0). The initial comparison with Cell Ranger showed 95% valid barcodes and 54.30% sequencing saturation. The raw digital gene expression matrix (UMI counts per gene per cell) from feature barcodes was filtered and normalized using the R package Seurat (version 3.1.1). Overall, genes detected in fewer than one percent of cells were filtered out, and cells were removed if they expressed fewer than 500 detected genes (UMI < 500) and had a percentage of mitochondrial DNA-derived genes expressed <25%. The Doublet Finder package (version 2.0.3) was also used to remove doublets. The scRNA-seq data that met quality control standards were used for transcriptomics analysis.

Unified manifold approximation and projection (UMAP) analysis was performed after removing low-quality cells and bimodal peaks, and the data were then normalized for expression using the “log normalize” method in the Seurat package, version 4.4.0. Principal component analysis (PCA) was then performed using the “Run PCA” function based on the normalized expression values. The top ten principal components (PCs) were selected from the PCA results using the Jackstraw alternative test algorithm for subsequent unsupervised clustering and cluster analysis. Cells were clustered using the “Find Clusters” function with appropriate resolution, and two-dimensional visualization was obtained by UMAP. The “Find All Markers” function (Log2 FC ≥ 0.26 and *p*-value ≤ 0.01) was used as a clustering condition, and only genes expressed in more than 10% of the cells in the cluster were considered. Cell cluster identities were assigned by manual annotation using a combination of marker genes identified from the literature, the list of marker genes, and the gene ontology of the cell type. The expression of the selected genes was plotted using functions like Feature Plot, Vln Plot, and Dot Plot in Seurat.

### 4.7. Data Statistics

After preliminary sorting of cell count and viability detection data using Excel 2016, an independent sample *t*-test was performed for statistical analysis using SPSS 25.0 software. The experimental results were expressed as the mean and the mean standard error.

## 5. Conclusions

Both scRNA-seq and snRNA-seq are powerful techniques for resolving cellular and molecular heterogeneity in animal tissues, yet each has distinct advantages depending on the experimental context. Direct comparisons of superiority are inappropriate as their performance depends critically on tissue type and experimental objectives. Whereas scRNA-seq excels in high-resolution cellular heterogeneity analysis, snRNA-seq is preferable for mechanically challenging tissues and archived frozen specimens. In the caprine pancreas, scRNA-seq demonstrates superior performance in both cell type annotation and the detection of functionally critical genes, such as pancreatic secretion and digestive enzymes. Therefore, under our experimental conditions, scRNA-seq emerged as the more suitable method for caprine pancreatic studies, given its enhanced cell type resolution and gene detection sensitivity. However, the choice between scRNA-seq and snRNA-seq should consider sample compatibility, technical differences, and experimental objectives. Further research should optimize tissue processing protocol and systematically evaluate how pre-analytical variables, including dissociation time and cold ischemia, influence sequencing outcomes, thereby establishing evidence-based selection criteria.

## Figures and Tables

**Table 1:** The results of sequencing and mapping.

Item	Treatment	
	scRNA	snRNA
Valid barcodes, %	96.70	96.40
Valid unique molecular identifiers, %	100.00	100.00
Fraction reads in cells, %	86.70	53.00
Sequencing saturation, %	49.40	60.60
Reads mapped to genome, %	86.20	93.70
Reads mapped antisense to gene, %	0.70	24.20
Total genes detected, n	20,346	23,626

**Table 2:** Statistics of goat pancreas scRNA-seq and snRNA-seq cell types.

Treatment	Item		
	Cell Type	Cell Number	Percentage (%)
scRNA	PSC_cell	5850	63.09
	AC_cell	1519	16.38
	Beta_cell	754	8.13
	EC_cell	269	2.90
	Alpha_cell	241	2.60
	DC_cell	193	2.08
	IC_cell	261	2.81
	Delta_cell	185	2.00
snRNA	Other	7300	56.18
	AC_cell	1493	11.49
	Beta_cell	1554	11.96
	EC_cell	1175	9.04
	Alpha_cell	1059	8.15
	DC_cell	211	1.62
	S_cell	202	1.55

**Table 3:** Statistics of digestive enzyme genes in scRNA-seq and snRNA-seq pancreatic acinar cells.

Item	Treatment	
	scRNA	snRNA
*CEL*	135.36	37.73
*CPB1*	34.22	23.31
*CTRC*	55.45	6.74
*PNLIPRP2*	45.56	7.65
*PRSS2*	431.7	11.77

## Data Availability

The datasets generated and analyzed during the current study are available in NCBI’s Gene Expression Omnibus, accessible via GEO serial login number GSE207644 (https://www.ncbi.nlm.nih.gov/geo/query/acc.cgi?acc=GSE207644, accessed on 11 November 2024).

## Supplementary Materials

The following supporting information can be downloaded at: https://www.mdpi.com/article/10.3390/ijms26083916/s1.

## Abbreviations

The following abbreviations are used in this manuscript:

AC_cell	Acinar cells
DAPI	4’-6-diamidino-2-phenylindole
DC_cell	Ductal cells
EC_cell	Endothelial cells
FBS	Fetal bovine serum
GEM	Ingle-cell gel-emulsion of beads
IC_cell	Immune cells
PCA	Principal component analysis
PCs	Principal components
PSC_cell	Stellate cells
S_cells	Stem cells
scRNA-seq	Single-cell RNA sequencing
snRNA-seq	Single-nucleus RNA sequencing
tSNE	T-distributed stochastic neighbor embedding
UMAP	Unified manifold approximation and projection
